# A Manual of Animal Vaccination

**Published:** 1887-01

**Authors:** 


**A Manual of Animal Vaccination,** preceded by considerations on vaccination in general. By Dr. E. Warlomont, Member of the Royal Academy of Medicine, of Belgium ; Founder of the State Vaccine Institute, Belgium, etc. Translated and edited by Arthur J. Harris, M. D., Senior Assistant Physician and Joint Lecturer to St. John's Hospital for Diseases of the Skin ; Consulting Physician to the Association for the Supply of Pure Vaccine Lymph. With an appendix showing the results of re-vaccination, and the comparative utility of animal vaccine. Philadelphia: John Wyeth & Brother, 1412-1416 Walnut street. 1886.

In this work of about 150 pages, Dr. Warlomont presents a most excellent resumé of the existing knowledge on the subject of vaccine and vaccination. He adds to this, his own vast experience on this subject. He records the comparitive results with animal and humanized virus, and proves conclusively the superiority of theformer over the latter. The chapter on vaccino-syphilis is very valuable.

